# Assessment of Potential Toxicity of Onion-like Carbon Nanoparticles from Grilled Turbot *Scophthalmus maximus* L.

**DOI:** 10.3390/foods11010095

**Published:** 2021-12-30

**Authors:** Zuzhe Wang, Jingran Bi, Haitao Wang, Mingqian Tan

**Affiliations:** 1 School of Food Science and Technology, Dalian Polytechnic University, Qinggongyuan 1, Ganjingzi District, Dalian 116034, China; wangzuxian1@126.com (Z.W.); bijingran1225@foxmail.com (J.B.); wanght@dlpu.edu.cn (H.W.); 2 Dalian Blue Peptide Technology Research & Development Co., Ltd., Dalian 116085, China

**Keywords:** mesoporous nanocarbon, nano-impact, food borne, nanotoxicology

## Abstract

Although the presence of foodborne nanoparticles was confirmed in grilled fish in a previous study, the evaluation of potential health risks of these NPs was insufficient. In the present study, the potential toxicity of onion-like carbon nanoparticles (OCNPs) separated from grilled turbot *Scophthalmus maximus* L. was evaluated using mouse osteoblasts cells model and zebrafish (*Danio rerio*) model. Cytotoxicity evaluation revealed that the OCNPs penetrated into the MC3T3-E1 cells without arousing cell morphology changes. No evident apoptosis or damage of cells was observed with increasing OCNPs’ concentration to 20 mg/mL. In the hemolysis test, OCNPs did not show an obvious hemolysis effect on red blood cells. In the acute toxicity test, the LC_50_ value (212.431 mg/L) of OCNPs to zebrafish showed a weak acute toxicity. In subacute toxicity test, after exposure to OCNPs (30 mg/L, 40 mg/L) for 10 days, a significant increase of reactive oxygen species level of zebrafish was observed. Meanwhile, redundant ROS content caused inhibition to several antioxidant enzymes and induced lipid and protein peroxidation damages according to the upregulation of malondialdehyde and protein carbonyl levels. The chronic toxicity test results indicated that oxidative stress was only observed in the high concentration group of OCNPs-treated zebrafish.

## 1. Introduction

In recent years, the utilization of nanoparticles (NPs) produced artificially has been explored in the food industry due to their distinct physicochemical properties [1,2,3]. On the other hand, NPs spontaneously generated during intense food processing have also drawn great interest in their characteristics and potential health risks. Compared to those artificially synthesized NPs used in food packaging, storage, and tracking [4], exposure of NPs that exist in daily food to humans is considered more frequent. For instance, the presence of carbon NPs was confirmed in several foods including bread, sugar caramels, and corn flakes [5]. In 2019, Zhao et al. [6] reported the presence of fluorescent NPs from roasted pork and their influence on locomotion behaviors and lifespans in *Caenorhabditis elegans*. Moreover, studies on carbon dots (CDs) from commercial beer revealed that a high dose of CDs could affect cell cycle progression and cause cell apoptosis of mouse osteoblasts cells (MC3T3-E1) [7]. Accordingly, although foodborne NPs are commonly considered to be not harmful, the existence of NPs in what range of daily consumed food is upon our expectation, and they may cause considerable health risks to organisms. In addition, the interaction of NPs from mature vinegar to dopamine and the interaction of NPs from grilled duck to human serum albumin revealed that foodborne NPs may have physiological implications by binding to certain substances in organisms [8,9]. However, the knowledge of the characteristics and potential toxicity of foodborne nanostructures remains insufficient. In previous research, the cytotoxicity of metal-based NPs, such as copper oxide NPs and zinc oxide NPs, was attributed to the substantial generation of reactive oxygen species (ROS), which may interrupt the antioxidant system in organisms [10,11]. Despite the difference in size, surface groups, and optical properties between metal-based NPs and carbon-based NPs, there is a possibility that foodborne carbon-based NPs induce excessive production of ROS as well. Therefore, studies on the impact of foodborne NPs on ROS and antioxidant system-related biomarkers may help us to get insights into the toxic mechanism of foodborne NPs. Furthermore, the toxicity evaluation of foodborne NPs mostly focused on cytotoxicity in vitro or acute toxicity in vivo [12,13]. Considering the broad presence of NPs in daily food, the foodborne NPs could easily be absorbed by the gastrointestinal tract; however, little work on that has been done. Research on subacute and chronic toxicity and metabolism process of foodborne NPs in organisms is in urgent need.

Grilled fish is a widely consumed and favored dish because of its good flavor and soft texture. Since the formation of foodborne NPs has been confirmed in complex physicochemical reactions of carbonaceous compounds during thermal treatment [14], it is reasonable to predict the existence of NPs in grilled fish. In 2018, Bi et al. [15] first reported the presence and formation mechanism of carbonate NPs from grilled pike eel *Muraenesox cinereus*, although toxicity assessment of those nanostructures is still unclear. Onion-like carbon particles (OCNPs) from the grilled turbot *Scophthalmus maximus* L. have been reported in our previous work, with an outer diameter in the range of 10–20 nm and a shell thickness of 3–5 nm. The potential toxicity of OCNPs to organisms drew our interest because of their specific hollow structure of OCNPs, which are different from normal carbonate NPs [16]. Many works have been conducted in understanding the potential risks of foodborne NPs, but little information is available about their subacute and chronic toxicity for foodborne hollow nanostructure like OCNPs. These foodborne NPs may be used as nanocarriers in the delivery of metal ions, including Zn^2+^, Ca^2+^, Fe^2+^, and so on, due to their small size and good water solubility [17,18,19]. The bioeffect of OCNPs is barely studied and their toxicity is probably highly related to their structures, including size and morphology [20]. Despite non-toxic foodborne nanoparticles causing minor oxidative stress, the in vivo evaluation of long period of exposure to OCNPs has not been reported. The novelty of this study is to investigate the cytotoxicity, acute toxicity, subacute toxicity, and chronic toxicity of the OCNPs produced from grilled turbot for the first time. The cytotoxicity of OCNPs was evaluated by measuring relative cell viability, cell membrane integrity, and the ROS level of MC3T3-E1 cells after incubation with OCNPs. The biocompatibility of OCNPs to red blood cells was investigated by a hemolysis test. Acute toxicity, subacute toxicity, and chronic toxicity characteristics of OCNPs were studied using a zebrafish model.

## 2. Materials and Methods

### 2.1. Materials

Ethyl acetate, ethanol, acetic acid, and sodium chloride were purchased from Tianjin Damao Chemical Reagent Co., Ltd. (Analytical grade, Tianjin, China). Bicinchoninic Acid (BCA) Protein Assay Kit (P0012) was bought from Shanghai Biyunlantian Biotechnology Co., Ltd. (Shanghai, China). Peroxidase (POD) Assay Kit (A084-1), Catalase (CAT) Assay Kit (A007-2), Glutathion Peroxidase (GSH-Px) Assay Kit (A005), Total Superoxide Dismutase (SOD) Assay Kit (A001-1), Malondialdehyde (MDA) Assay Kit (A003-1), Reactive Oxygen Assay Kit (E004), Lactate Dehydrogenase (LDH) Assay kit (A020-2), Reduced Glutathione (GSH) Assay Kit (A006-1), and Protein Carbonyl (PCO) Content Assay Kit (A087) were purchased from Nanjing Jiancheng Bioengineering Institute (Nanjing, China).

The cell line of MC3T3-E1 was provided from Cell Resource Center, Shanghai Institute of Biological Sciences, Chinese Academy of Sciences. Fetal bovine serum (F8245) was bought from Hangzhou Sijiqing Biological Engineering Materials Co., Ltd. (Hangzhou, China). Trypsin-EDTA digestive juice (0.25%) and DMEM buffer (32571093) were purchased from Thermo Fisher Scientific Inc. Co., Ltd (Shanghai, China). Hank’s Balanced Salt Solution (HBSS, SH30588.01) buffer and dimethyl sulfoxide were purchased from Shanghai Yisheng Biotechnology Co., Ltd. (Shanghai, China). 3-(4,5)-dimethylthiahiazo (-z-y1)-3,5-di- phenytetrazoliumromide (MTT) Cell Proliferation and Cytotoxicity Assay Kit (C009) was purchased from Shanghai Biyuntian Biotechnology Co., Ltd. (Shanghai, China).

Adult zebrafish of the same batch and same age were used as the testing materials. In order to ensure the consistency of the test conditions and the credibility of the test results, the domesticated conditions were consistent: natural photological period, laboratory temperature 23 ± 1 °C, the water used for the toxicity test of zebrafish was deionized water aerated for 24 h, water temperature was 23 ± 1 °C, water quality was pH 7.8 ± 0.2, dissolved oxygen > 70%. After 14 days of domestication, the death rate was less than 10%. Food was not provided for the zebrafish 24 h before the test and during the test. Healthy zebrafish with body length of 2–3 cm and body weight of 0.15–0.25 g were randomly selected as experimental materials.

### 2.2. Extraction of OCNPs from Grilled Turbot

In previous study, carbonaceous nanostructures could be extracted from roasted pike eel [15]. Fresh turbot raw material of 1000 g was cut into 3 × 3 × 1.5 cm pieces, and grilled in an oven at 230 °C for 30 min. The grilled samples were transferred to ethanol (*w*:*v* = 1:20) and stirred for 24 h. After removal of the precipitate, the ethanol solution was evaporated and 15 mL water was added. The sample was then extracted with about 45 mL of dichloromethane three times to remove the liposoluble substance. The water phase was then dialyzed with 1000 Da molecular cut-off dialysis bag for 48 h against deionized water. The dialysate was then collected and vacuum-freeze-dried to yield solid powder with a production yield of about 1.6%. OCNPs obtained were dispersed in deionized water and observed under transmission electron microscope (TEM, JEM-2100, JEOL, Tokyo, Japan), then the size of OCNPs on TEM images was calculated by ImageJ bundled with 64-bit Java software (National Institutes of Health, Bethesda, MA, USA).

### 2.3. Bio-Distribution of OCNPs in MC3T3-E1 Cells

The OCNPs from grilled turbot were dispersed in 10% fetal bovine serum in DMEM with a final concentration of 1.5 mg/mL as OCNPs medium. Thereafter, MC3T3-E1 cells digested by 0.25% trypsin in EDTA were seeded in a plate at a density of 1 × 10^5^ cells per well. To each well was added 400 μL of OCNPs medium, which was then incubated for 24 h in 5% CO_2_ at 37 °C. After incubation, the cells were washed with HANKS balanced salt buffer solution (HBSS) buffer three times (500 μL each time), then part of the HBSS buffer was examined under a laser confocal microscope (SP8, Leica, Wetzlar, Germany) for cell imaging. Wavelengths of 405, 488, and 543 nm were used to excite the fluorescence of OCNPs.

### 2.4. Cytotoxicity of OCNPs

MC3T3-E1 cells seeded in 96-well plate at a density of 5.0 × 10^3^ cells per well were incubated with different concentrations (0, 0.31, 0.63, 1.25, 2.5, 5.0, 10.0, and 20.0 mg mL^−1^) of OCNPs at 37 °C with 5% CO_2_ atmosphere for 24 h. The cell plate was added 20 μL of 5 mg/mL of MTT solution and incubated for 3 h, followed by removing the supernatant, adding 100 μL DMSO solution, and shaking for 10 min. Then an Infinite F200 PRO microplate reader (Tecan Ltd., Salzburg, Austria) was used to record the absorbance value of each well at 490 nm. The cell viability was calculated by the equation of cell viability (%) = OD eg/OD cg × 100%, where OD eg is the absorbance of the cells with OCNPs in the experimental group and OD cg is the absorbance of the cells without OCNPs in the control group.

The cell membrane integrity was checked by the LDH assay kit. Relative LDH release ratio of MC3T3-E1 cells incubated with OCNPS was evaluated using LDH Test Kit to check the cell membrane integrity. The ROS level of MC3T3-E1 cells treated with OCNPs was evaluated with ROS Test Kit.

### 2.5. Hemolysis Effect of OCNPs

Fresh blood from adult Kunming mice was collected in a 2 mL Eppendorf tube, and centrifuged at 3000 rpm/min for 5 min below 4 °C. After removal of supernatant, the red blood cells pellet was diluted with PBS buffer. In the experimental group, 0.2 mL of different concentrations (1, 5, 10, 20 mg/mL) of OCNPs were diluted to 0.8 mL with PBS buffer and added 0.2 mL red blood cells. A total of 0.8 mL ultra-pure water and PBS buffer were added to 0.2 mL red blood cells as negative and positive control, respectively. Solutions were kept for 0.5 h and centrifuged at 3000 rpm/min for 5 min below 4 °C. The UV absorbance value at 541 nm of the supernatant was detected and the hemolysis ratio of red blood cells was calculated.

### 2.6. Acute Toxicity Evaluation

Based on OECD 203 [21], the 50% lethal concentration (LC_50_) of OCNPs to zebrafish was calculated according to LC_0_ value and LC_100_ value using a static exposure test. Healthy zebrafish were randomly picked and divided into seven groups, and fed in a 1.5 L exposure solution with different concentration (25, 50, 100, 250, 500, 750, or 1000 mg/L) of OCNPs. All concentration groups were performed in triplicates. The poisoning sign and the number of deaths were recorded constantly within early 6 h, and at 24, 48, 72, or 96 h. Dead individuals were removed from the tank immediately for keeping the tank from pollution. The criterion of the death of zebrafish was established based on signs including no opercular movement, non-reaction to external stimulus, and losing swimming ability. According to Karber’s method, LC_50_ value and 95% confidence limit were calculated using the following equation:

LC_50_ = lg^−1^[Xm − i (∑P − 0.5)] (1)

LC_50_’s 95% confidence limit = log^−1^(X_50_ ± 1.96Sx_50_) (2)

where Xm is the logarithm value of the maximum dose, i is the logarithm value of the ratio of two adjacent doses, P is the death ratio of animals in each group, ∑P is the sum of the death ratio of animals in each group, n is the animal’s number of each group, SX_50_ is the standard error of logLC_50_, X_50_ equal logLC_50_. Safe concentration (SC) was calculated by the equation of SC = LC_50_ × 0.1. According to the test method for fish acute toxicity for dangerous chemical products, acute toxicity of OCNPs was graded based on hazard ranking of acute toxicity as in Table 1.

### 2.7. Subacute Toxicity Evaluation

Based on OECD 204 (1984) [22], a semi-static toxicity test was adopted, namely half of the exposure solution was renewed at each 48 h during the test. Referring to the results in the acute toxicity test, healthy zebrafish were randomly picked and divided into 6 groups, and fed in a 1.5 L exposure solution with different concentrations (0, 5, 10, 20, 30, or 40 mg/L) of OCNPs. Three random individuals were picked at 1, 3, 7, 10, and 14 days after exposure. Then, the fish were executed, washed with cold 0.9% NaCl solution, and homogenized with 9 times volume of physiological saline. The supernatant was collected after 10 min centrifugation at 3500 r/min and preserved at low temperature as 10% enzyme containing supernatant for further tests.

A BCA protein assay kit was used for protein quantification of enzyme containing supernatant from test fish. To evaluate the ROS level in 10% enzyme containing supernatant, 190 μL of supernatant was mixed with 10 μL of DCFH-DA (10 μM) at 37 °C water bath for 30 min. The fluorescent absorbance value was measured using excitation at 500 nm and emission at 525 nm with a fluorescence detector. A total of 0.1 mL supernatant was used for MDA quantification according to MDA assay kit instruction. The supernatant with a volume of 450 μL was used for PCO quantification according to PCO assay kit instruction.

As for oxidative stress evaluation, 10% enzyme containing supernatant was diluted to one-fiftieth of the original concentration, 50 μL of the diluted solution was used for SOD concentration evaluation according to SOD assay kit. A total of 0.1 mL 10% enzyme containing supernatant was used for POD concentration evaluation according to POD assay kit. The supernatant containing 10% enzyme was diluted to one-twentieth of the original concentration, 50 μL of the diluted solution was used for CAT concentration evaluation according to the instructions of the CAT assay kit. GSH-Px concentration was evaluated according to the instructions of the GSH-Px assay kit.

### 2.8. Chronic Toxicity Test

Healthy zebrafish were randomly picked and divided into three groups, and fed in a 1.5 L exposure solution with different concentrations (2.5, 5, 10 mg/L) of OCNPs for 30 days. Enzyme-containing supernatant was collected using the same method as in Section 2.7. Methods of protein quantification and testing of ROS, MDA, PCO, SOD, POD, CAT, GSH-Px were performed as mentioned in Section 2.7.

### 2.9. Statistics Analysis

Statistical product and service solutions (SPSS) statistics 19 package (IBM, Corp., Armonk, NY, USA) was used for statistical analysis between exposure group and control group by one-way analysis of variance (*p* < 0.05, *p* < 0.01).

## 3. Results

### 3.1. Preparation of OCNPs

**Figure 1:**
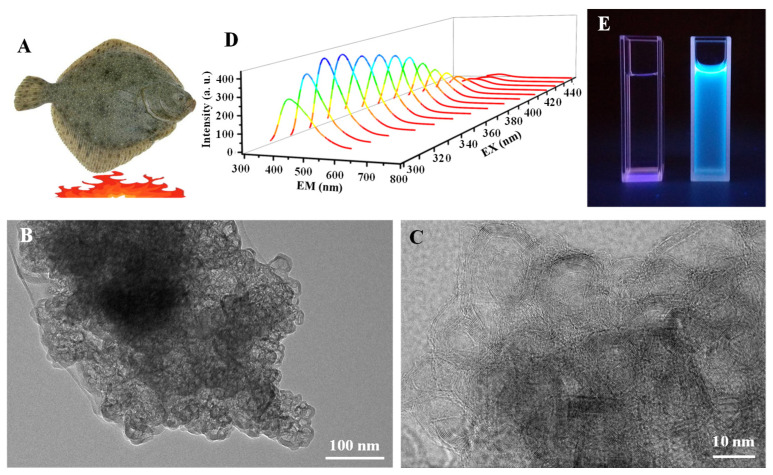
(**A**) Scheme illustration of grilled turbot at 230 °C. TEM images of (**B**) onion-like carbon particles (OCNPs) and the enlarged TEM image (**C**) of OCNPs derived from grilled turbot, (**D**) fluorescence spectra and (**E**) fluorescence photograph of the OCNPs (right) using water (left) as a control.

The onion-like carbon particles (OCNPs) were reported in our previous work with small size and a hollow structure [23]. The OCNPs were extracted from turbot flesh grilled at 230 °C for 30 min, which showed an onion-like structure with multilayers as displayed in Figure 1B,C. The inner and outer diameter size of the OCNPs was 12.26 ± 5.48 nm and 16.49 ± 5.95 nm, respectively (Figure S1), with a multilayer shell about 3–5 nm (Figure 1C), which was much larger than the common size of carbon NPs [24]. Moreover, the OCNPs emitted fluorescence with a maximum emission from 370–500 nm under the excitation of 300–450 nm (Figure 1D), indicating a typical fluorescence emission behavior of carbon NPs. Strong fluorescence could be observed when the OCNPs were exposed to ultraviolet light (Figure 1E). The small size and onion-like structure with strong fluorescence of the OCNPs can probably enter our body when they are taken with food, which has attracted great attention for their in vitro and in vivo impacts when they are present in a daily meal.

### 3.2. Cytotoxicity of OCNPs in MC3T3-E1 Cells

**Figure 2:**
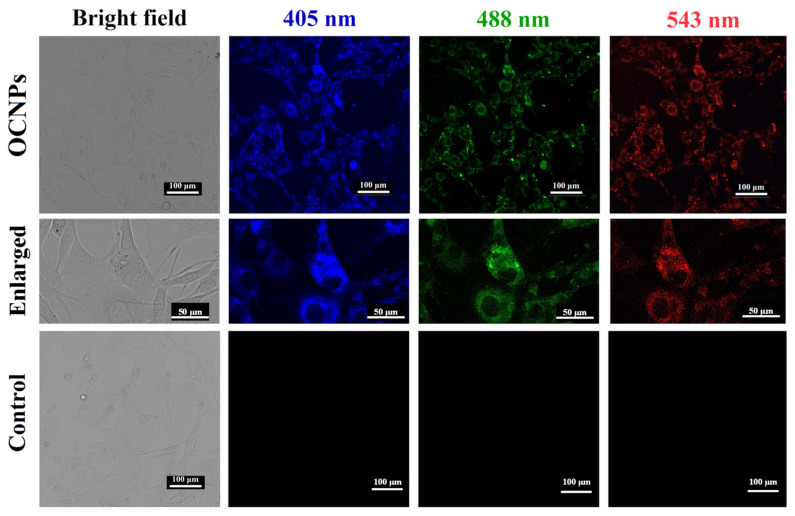
Images of bright field and fluorescence images of MC3T3-E1 cells incubated with OCNPs from grilled turbot.

Previous studies implied that carbonate NPs, including carbon dots and carbon nanotubes, could be use as biocompatible nanoprobes since they can be absorbed by cells in vitro [25,26]. For this reason, the bio-distribution and cytotoxicity of OCNPs from grilled turbot were investigated in living cells. Given that the MC3T3-E1 cells showed no fluorescence under a laser scanning confocal microscope (Figure 2), the location of OCNPs can be tracked if OCNPs enter the cells due to their unique fluorescence property. The fluorescence of MC3T3-E1 cells indicated that OCNPs penetrated the MC3T3-E1 cell membrane and distributed evenly in the cytoplasm (enlarged images in Figure 2). However, due to the surface chemistry compared to other foodborne nanoparticles reported, aside from the slightly weak dispersibility, the OCNPs were blocked outside of the nucleus. In contrast to the control group, no cellular morphology change derived from OCPNs’ entry was observed. Meanwhile, blue, green, and red fluorescence of OCPNs could be observed in the MC3T3-E1 cells by different excitation wavelength of 405, 488, and 543 nm, respectively, which was consistent with the excitation-dependent fluorescence behavior of carbonate NPs [27]. Therefore, the internalization of OCNPs into the living cells indicated that the bio-influence of the nanostructure could not be ignored, and further study should be carried out in understanding their interaction and nanoimpact on organisms.

**Figure 3:**
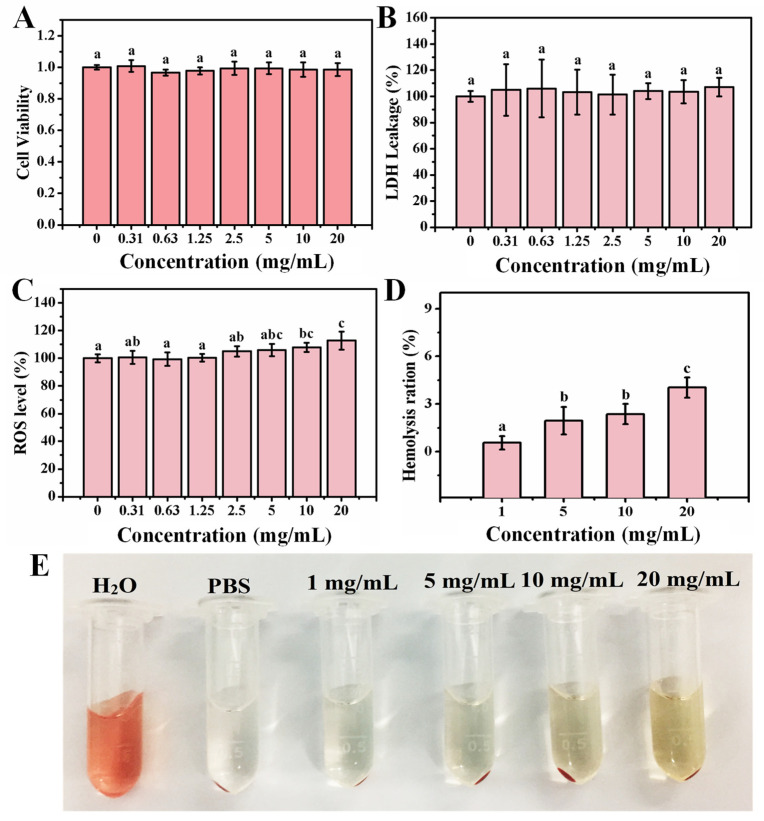
(**A**) Relative cell viability of MC3T3-E1 cell, (**B**) relative LDH release level of MC3T3-E1 cell, (**C**) relative ROS values of MC3T3-E1 cell after incubation with different concentrations OCNPs from the grilled turbot for 24 h. (**D**) Hemolysis ratio of red blood cells, (**E**) changes of red blood cells after incubation with different concentrations OCNPs for 1 h. Different letters above columns indicate significant differences (*p* < 0.05) between treatments.

The OCNPs from grilled turbot accumulated in MC3T3-E1 cells and their effect on cell viability was then investigated. As shown in Figure 3A, the relative cell viability of MC3T3-E1 cell treated with OCNPs demonstrated that the cell viability was about 100%, and no significant toxic effect was found when the concentration of OCNPs was below 20 mg/mL as compared with the control group (*p* > 0.05). However, our previous work showed that the carbon dots extracted from cigarettes smoke reduced cell viability of HepG2 cells in a dose-dependent manner [28]. The relative cell viability of MC3T3-E1 cells remained 91.63% even when the concentration of OCNPs reached 20 mg/mL. This result indicates that good cellular biocompatibility and almost no cytotoxicity of OCNPs was observed in vitro even at such high concentration of the nanoparticles. Moreover, the release level of lactate dehydrogenase (LDH) from cells was measured to evaluate membrane integrity after OCNPs treatment [29]. Moreover, as displayed in Figure 3B, no significant increase of LDH level was detected (*p* > 0.05) with the increase of OCNPs’ concentration, indicating that there was no membrane damage due to severe cell apoptosis and necrosis after OCNPs treatment. A high level of LDH level in living cells indicated a sign of cellular damage, mainly in injuries that affect membrane integrity. The OCNPs did not cause visible damage to the lipid bilayer of the cellular membrane and there was no detectable lactate dehydrogenase leaked from the living cells. This might be due to the good safety property of the OCNPs upon internalization by MC3T3-E1 cells.

The MC3T3-E1 cells were applied for the impact of hydroxyapatite NPs on ROS generation and the activity of SOD and GSH-Px, whereas the oxidative-induced lysosomal and mitochondrial damage was reported [30]. The OCNPs with small size and high surface-to-volume ratio might be a potential production matrix for ROS that can cause damage to biological molecules such as DNA, lipids, cholesterol, and protein in the organism. To clarify the impact of OCNPs to ROS level in living cells, relative ROS values of MC3T3-E1 cell after incubation with OCNPs were investigated. Compared with the control group, the ROS values in the experimental groups with OCNPs concentrations in the concentration of 0–5.0 mg/mL did not show significant alteration (*p* > 0.05), which was in agreement with the effect of the same concentrations of OCPNs to cell viability. However, when the concentration of OCNPs was more than 10.0 mg/mL, the ROS values of MC3T3-E1 cells significantly increased. The results indicated that the OCNPs at concentrations below 5.0 mg/mL would not disturb the balance between generation and elimination of ROS in MC3T3-E1 cells but might cause oxidative stress at concentrations over 10.0 mg/mL. Although the ROS level elevated with the increase OCPNs concentration, the ROS effect on the organisms requires further studied by other methods.

Since red blood cells will inevitably be exposed to NPs in the case of NPs entering into organisms, investigation into the hemolysis effect of OCNPs on red blood cell is quite necessary. If the OCNPs possess low compatibility, they may cause severe hemolysis, and hemoglobin will largely leach from the cells and increase the UV absorbance value at 541 nm. In this study, after 1 h incubation with OCNPs, the hemolysis ratio of red blood cells gradually increased. With the concentration of OCNPs exceeding 20 mg/mL, hemolysis ratio was only 4.03%. Moreover, based on the photographs of the red blood cells incubated with OCNPs, the supernatant was not obviously red (Figure 3E), further indicating that the OCNPs at concentrations below 20 mg/mL had a minor impact on hemolysis and were bio-compatible with red blood cells. The concentration of 20 mg/mL for OCNPs is an extremely high level for the nanoparticles in blood, and regardless, grilled meat with a high concentration of OCNPs on the food surface should be paid more attention when they are eaten. In this case, according to the results of these cytotoxicity tests, the OCNPs from grilled turbot did not appear evident in vitro cytotoxicity; further in vivo toxicity tests were needed consequently.

### 3.3. In Vivo Toxicity Evaluation Using Zebrafish Model

**Figure 4:**
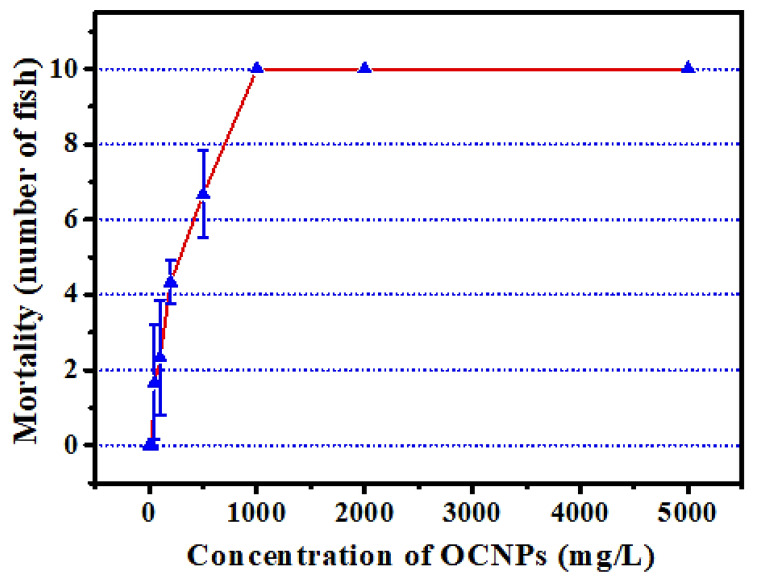
Effect of different concentration OCNPs on the mortality of Zebrafish.

Since the OCNPs can be easily internalized into cells, they might induce health risks after a short or long period of accumulation in vivo. Therefore, to comprehensively understand the impact of OCNPs from grilled fish to organisms, in vivo toxicity tests are necessary for more toxicity profiles. To this end, zebrafish was selected as a toxicological model for rapid in vivo tests, which had been used in a toxicology study because of their human-like genome, ease of breed, short life cycle, and high-throughput chemical screening [31]. Fish is extremely sensitive to exogenous stimulus, and a toxin in the living environment will cause a series of poisoning reactions. Thus, zebrafish acute toxicity test is really useful for evaluating the impacts of OCNPs to organisms. The mortality test of zebrafish treated with OCNPs for 96 h revealed that the maximal tolerance dose (LC_0_) and absolute lethal concentration (LC_100_) value for zebrafish after exposure to OCNPs were 25 mg/L and 1000 mg/L, respectively (Figure 4). In contrast, silver nanoparticles possess almost same level of LC_0_ value at 25–50 mg/kg and relatively lower LC_100_ value at 500–1000 mg/kg [32]. Therefore, the silver nanoparticles were more harmful than OCNPs for zebrafish. It is indicated that the toxicity of NPs is highly related to the shape and size of themselves. The OCNPs can be easily excreted by cells due to its relatively small size. Our data showed that 96 h LC_50_ value for zebrafish with OCNPs treatment was at 212.43 mg/L with a safe concentration (SC) of 21.24 mg/mL, which was located at weak toxicity range in Table 2 calculated based on LC_0_ and LC_100_ value via Karber’s method.

**Figure 5:**
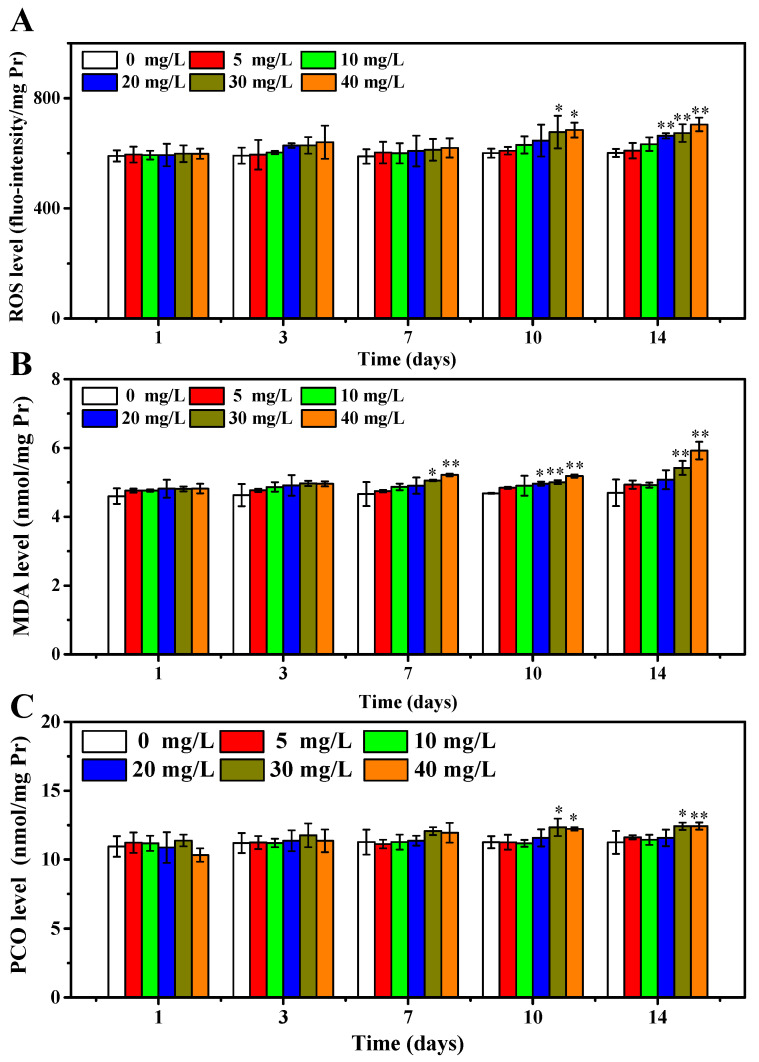
(**A**) ROS level of zebrafish, (**B**) MDA concentration of zebrafish, (**C**) PCO concentration of zebrafish after exposure to OCNPs in subacute toxicity test. *, ** indicate significant differences (*p* < 0.05) and remarkably significant differences (*p* < 0.01) among treatments.

At present, the hypothesis on the toxicity mechanism of NPs is mostly focused on the speculation that NPs disorder the ROS level in organisms, then cause oxidative damage to lipids and proteins and eventually induce harmful disease. According to the results from the acute toxicity test, the zebrafish were cultured with OCNPs at concentrations of 0, 5, 10, 20, 30, and 40 mg/L, and randomly tested at 1, 3, 7, 10, and 14 days. ROS level and biomarkers related to oxidative stress of test zebrafish were investigated to evaluate subacute toxicity of OCNPs. As shown in Figure 5A, the ROS level of the zebrafish body did not show a significant difference compared with the control group (*p* > 0.05) in the 5, 10 mg/L group from 1 to 14 days and in the 20 mg/L group from 1 to 10 days after exposure. However, the ROS level of the 20 mg/L group increased significantly after 14 days (*p* < 0.05). When the concentration of OCNPs increased to 30 mg/L and 40 mg/L, the ROS level remained constant for 7 days (*p* > 0.05), increased significantly after 10 days (*p* < 0.05), and increased remarkably after 14 days (*p* < 0.01). It is obvious that the impact of OCNPs on ROS level in zebrafish body intensified along with the increase of OCNPs concentration and exposure time.

In addition, as products of the lipid and protein oxidation process, the MDA and PCO levels in zebrafish body were investigated to evaluate oxidative damage of the OCNPs [33,34]. As shown in Figure 5B, the MDA concentration of zebrafish did not show a significant difference after 3 days of exposure (*p* > 0.05). After 7 days, the 30 mg/L group and the 40 mg/L group showed a significant difference (*p* < 0.05) and remarkable difference (*p* < 0.01), respectively. After 10 days treatment, the MDA concentration in the 20 mg/L group also increased significantly (*p* < 0.05), while MDA concentration in the 30 mg/L group and the 40 mg/L group increased remarkably (*p* < 0.01) and the increase trend continued to 14 days. Excessive ROS is suggested to cause lipid peroxidation, leading to damaging the normal structure of the cell membrane and intracellular fluid leak, then inducing organism damage and finally disease or death. Therefore, it was revealed that OCNPs caused substantial generation of ROS and lipid peroxidation inside the zebrafish body, and the increment of MDA content due to lipid peroxidation has a toxic effect to zebrafish.

The PCO content profile in zebrafish body after exposure to OCNPs is shown in Figure 5C. When the concentration of OCNPs was at 5, 10, 20 mg/L, the PCO concentration did not show a significant difference during whole exposure time (*p* > 0.05). However, the PCO concentration in the 30 mg/L group increased significantly after both 10 days and 14 days, while the PCO concentration in 40 mg/L group increased significantly after 10 days (*p* < 0.05) and remarkably after 14 days (*p* < 0.01). During oxidative stress conditions, the PCO values increased due to oxidative damage to proteins, thus causing disorder or inflammation in organisms. Considering the results of the impact of OCNPs to ROS level in zebrafish, it was suggested that a high level of ROS caused severe protein peroxidation.

**Figure 6:**
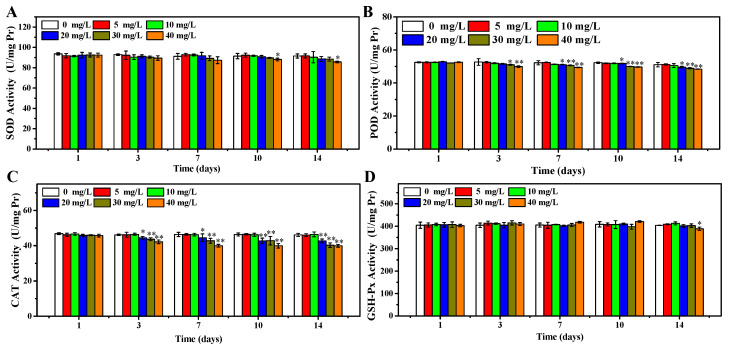
(**A**) SOD concentration of zebrafish, (**B**) POD concentration of zebrafish, (**C**) CAT concentration of zebrafish, (**D**) GSH-Px concentration of zebrafish after exposure to OCNPs in subacute toxicity test. *, ** indicate significant differences (*p* < 0.05) and remarkably significant differences (*p* < 0.01).

In accordance with the results above, the OCNPs from grilled turbot were confirmed to cause increment of ROS level and oxidative damage. Moreover, the effects of OCNPs on key enzymes of oxidative stress including SOD, POD, CAT, and GSH-Px were evaluated. These enzymes attempt to inhibit oxidative damage by triggering chemical reactions to get rid of free radicals. As shown in Figure 6A, the SOD concentration of zebrafish body remained at a close level in 0 to 30 mg/L group after 14 days exposure (*p* > 0.05). Meanwhile, in the 40 mg/L group, the SOD concentration significantly declined after 10 and 14 days compared to the control group (*p* < 0.05). It could be explained that SOD was inhibited due to oxidative damage in the zebrafish body via a certain time of accumulation of high level concentration of OCNPs. In addition, regarding the results of the impacts of OCNPs to MDA and PCO content in the zebrafish body, the reduction of SOD may further promote accumulation of free radicals and generation of ROS in late days of exposure, then consequently enhance oxidative damage to lipids and proteins. Moreover, this kind of inhibition from OCNPs also acted on GSH-Px (Figure 6D). As shown in Figure 6B,C, the POD and CAT concentration showed significant differences at earlier exposure time and with lower concentration of OCNPs compared to SOD and GSH-Px, indicating that OCNPs possessed an inhibition effect on POD and CAT. Thus, the inhibition impact of OCNPs to POD and CAT might fail to protect the generate-eliminate balance of hydrogen peroxide, thus causing organism damage.

### 3.4. Chronic Toxicity Test

**Figure 7:**
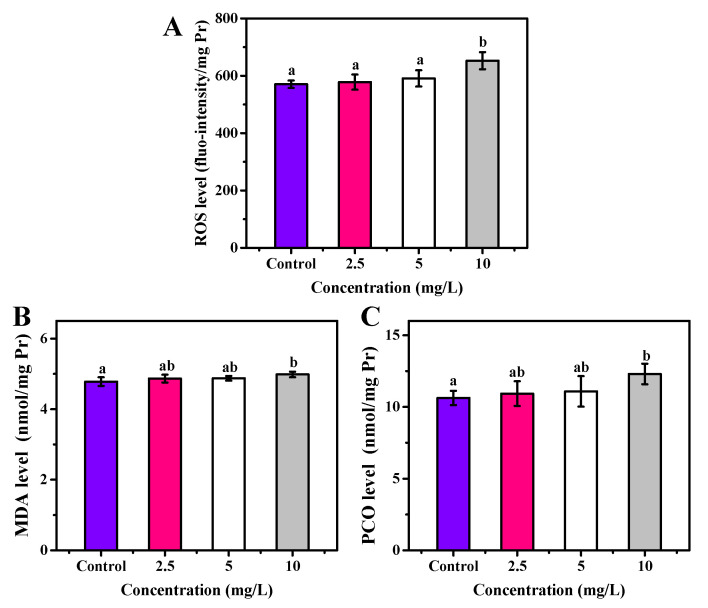
(**A**) ROS, (**B**) MDA, and (**C**) PCO level of zebrafish after exposure to OCNPs in chronic toxicity test. Different letters above columns indicate significant differences (*p* < 0.05) between treatments.

The OCNPs from grilled turbot exhibited non-negligible toxicity to zebrafish in the subacute toxicity test. Moreover, due to the special size effect, surface structure, and chemical properties of NPs, whether they caused chronic toxicity to organisms was of concern. Thus, investigation on long-term toxicity and metabolite process of OCNPs in organisms was highly required for evaluating their biosafety. After 30 days exposure to OCNPs at concentrations of 2.5, 5, or 10 mg/L, the ROS level of zebrafish was investigated. There was no significant change in ROS level with 2.5 and 5 mg/L OCNPs solution (*p* > 0.05) compared with the control group, indicating that the generate-eliminate balance of ROS still stayed at a low concentration of OCNPs in zebrafish (Figure 7A). With 10 mg/L of OCNPs treatment, the ROS level increased significantly (*p* < 0.05), which meant ROS had enriched to a level that was over the eliminate ability of the anti-oxidative system in zebrafish. Figure 7B,C illustrate MDA and PCO profiles, respectively, which imply the oxidative damage level of lipids and proteins in zebrafish after exposure. Similar to the results of ROS level, a significant increment of MDA and PCO concentration was only observed in the 10 mg/L groups (*p* < 0.05) in comparison with the control group. These results revealed that massive overdose of OCNPs would be toxic due to increment of ROS level surpassing the viability of zebrafish. Polyunsaturated fatty acids in cell membrane were oxidized, and the side chain amino acid of proteins was degraded, eventually greatly promoting MDA and PCO content.

**Figure 8:**
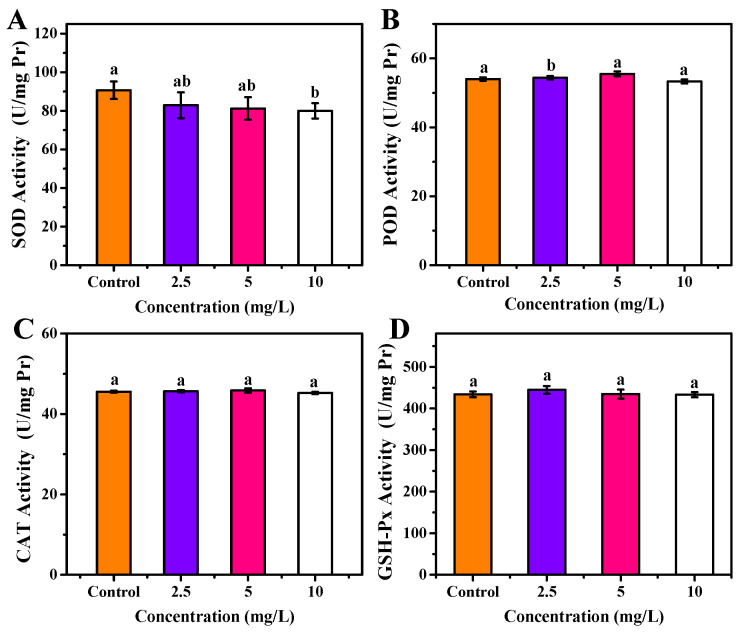
(**A**) SOD activity, (**B**) POD activity, (**C**) CAT activity, (**D**) GSH-Px activity of zebrafish after exposure to OCNPs in chronic toxicity test. Different letters above columns indicate significant differences (*p* < 0.05) between treatments.

As shown in Figure 8A, no significant change on SOD concentration in the zebrafish body was observed after 30 days of exposure to 2.5 and 5 mg/L OCNPs (*p* < 0.05), but 10 mg/L of OCNPs provided significant inhibition on SOD concentration in the zebrafish body (*p* < 0.05). The SOD is an enzyme that alternately catalyzes the dismutation (or partitioning) of the superoxide radical into ordinary molecular oxygen (O_2_) and hydrogen peroxide (H_2_O_2_). The level of SOD decreasing suggests that the by-product of oxygen metabolism probably increased when the fish was exposed to a higher concentration of OCNPs (10 mg/L).

Meanwhile, 2.5 mg/L OCNPs caused oxidative stress in the zebrafish body, resulting in a significant increase of POD concentration (*p* < 0.05) (Figure 8B). However, when OCNPs reached 5 mg/L, overdose of ROS prompted oxidative damage, thus, the POD concentration was significantly inhibited compared with 2.5 mg/L group (*p* < 0.05). Moreover, the CAT concentration and GSH-Px concentration did not differ from the control group in all test groups (Figure 8C,D).

## 4. Conclusions

In the current study, the toxicity of OCNPs separated from grilled turbot was evaluated, and the OCNPs from grilled turbot could easily enter MC3T3-E1 cells. There was barely evident damage to cells for OCNPs but a significant increase of ROS level with a concentration exceeding 20.0 mg/L. In the subacute toxicity and chronic toxicity tests, exposure to high concentrations of OCNPs caused ROS generation and oxidative damage to lipids and proteins and inhibited the antioxidant system in the zebrafish body. The mortality of zebrafish in the acute toxicity test might be due to the oxidative damages over their tolerance. In addition, the impact of OCNPs on zebrafish was reinforced with the increase of OCNPs concentration and exposure time. It is possible that the uptake of OCNPs for a prolonged time could induce considerable hazards to organisms.

## Figures and Tables

**Table 1:** Hazard ranking of acute toxicity.

96 h LC_50_ (mg/L)	<1	1–10	10–100	100–1000
Toxicity grade	severe	high	moderate	weak

**Table 2:** The 96 h LC_50_ values for zebrafish after exposure to OCNPs.

Material	96 h LC50 (mg/L)	95% Confidence Limit (mg/L)	Safe Concentration (SC) (mg/L)	Hazard
OCNPs	212.43	142.00~317.76	21.24	weak

## Data Availability

Not applicable.

## Supplementary Materials

The following are available online at https://www.mdpi.com/article/10.3390/foods11010095/s1, Figure S1: Histogram showing the inner (A) and outer (B) diameter size of the OCNPs.
