# Supplementary material for: Assessment of Potential Toxicity of Onion-like Carbon Nanoparticles from Grilled Turbot Scophthalmus maximus L
Source: Foods. 2021 Dec 30;11(1):95. doi: 10.3390/foods11010095 (PMC8749973; doi:10.3390/foods11010095)
Supplement: Supplementary file 1 [file foods-11-00095-s001.zip › foods-1504455-supplementary.pdf]

Supplementary Material

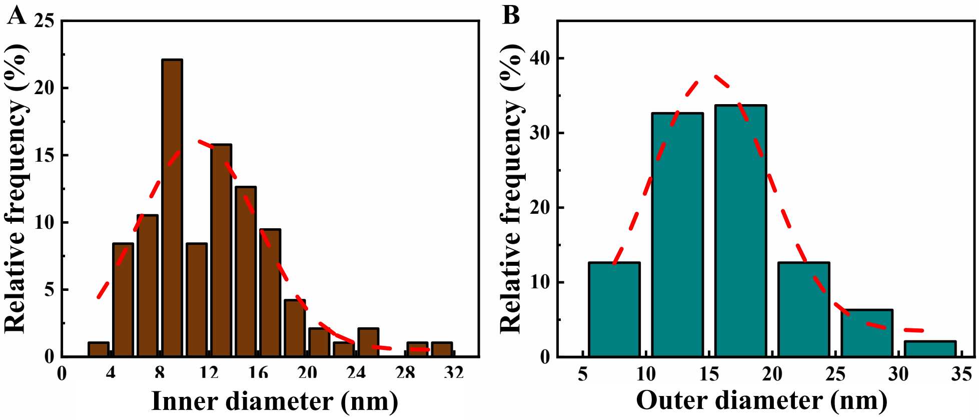

Figure S1. Histogram showing the inner (A) and outer (B) diameter size of the OCNPs.
